# Spatiotemporal Transcriptomic Atlas of Developing Embryos and Vegetative Tissues in Flax

**DOI:** 10.3390/plants11152031

**Published:** 2022-08-04

**Authors:** Peng Gao, Shuqing Qiu, Xingliang Ma, Isobel A. P. Parkin, Daoquan Xiang, Raju Datla

**Affiliations:** 1 Global Institute for Food Security, University of Saskatchewan, Saskatoon, SK S7N 4L8, Canada; 2 Saskatoon Research and Development Centre, Agriculture and Agri-Food Canada, 107 Science Place, Saskatoon, SK S7N 0X2, Canada; 3 Aquatic and Crop Resource Development, National Research Council Canada, 110 Gymnasium Place, Saskatoon, SK S7N 0W9, Canada; 4 Department of Plant Science, University of Saskatchewan, Saskatoon, SK S7N 5A8, Canada

**Keywords:** flax, transcriptome, development, embryogenesis, spatiotemporal landscape

## Abstract

Flax (*Linum usitatissimum* L.) is an important multipurpose crop widely grown for oil and fiber. Despite recent advances in genomics, detailed gene activities during the important reproductive phase of its development are not well defined. In this study, we employed high-throughput RNA-sequencing methods to generate in-depth transcriptome profiles of flax tissues with emphasis on the reproductive phases of five key stages of embryogenesis (globular embryo, heart embryo, torpedo embryo, cotyledon embryo, and mature embryo), mature seed, and vegetative tissues viz. ovary, anther, and root. These datasets were used to establish the co-expression networks covering 36 gene modules based on the expression patterns for each gene through weighted gene co-expression network analysis (WGCNA). Functional interrogation with Gene Ontology (GO) and Kyoto Encyclopedia of Genes and Genomes (KEGG) of dominantly expressed genetic modules in tissues revealed pathways involved in the development of different tissues. Moreover, the essential genes in embryo development and synthesis of storage reserves were identified based on their dynamic expression patterns. Together, this comprehensive dataset for developing embryos, mature seeds and vegetative tissues provides new insights into molecular mechanisms of seed development with potential for flax crop improvement.

## 1. Introduction

Flax (*Linum usitatissimum* L.), known as common flax or linseed, has been domesticated for ~7000 years, and this ancient crop has been increasingly valued in recent years for the highly valued health-promoting nutrients in the seeds [1]. Notably, flax seed contains dietary fiber and health promotive lignans [2,3] with a high ratio of alpha linolenic acid (ALA, an omega-3 fatty acid) and linoleic acid (LA, an omega-6 fatty acid), both of which are essential fatty acids for human health [4]. Canada’s Food Guide recommends flax seed as a healthy food that could decrease cholesterol levels and reduce the risk of cardiovascular disease [4,5]. Additionally, the stem fiber of flax has been widely used for highly valued linen for clothing and in the manufacturing industry [3].

Flax is a self-pollinating diploid plant containing 30 chromosomes with an estimated genome size of ~370 Mb. The floral dip method has been well established for flax transformation [6], making this crop amenable for genetic improvement and application of gene editing technologies. The availability of the flax genome sequence offers new opportunities for genetic dissection of important traits and improvement of this ancient crop [7]. For example, the transcriptome analysis of flax under edaphic stress [8], drought tolerance [9,10] and phloem fiber synthesis [11] have been investigated and advanced knowledge and genomics tools have been generated. These genome-wide analyses also provide molecular evidence for studies on the important flax-seed development-related traits from the gene expression level or the structural variance, including the regulation of the cyclolinopeptides in flax seed and the identification of candidate genes for flax-seed fatty-acid metabolism [12,13]. On the other hand, several studies have investigated the gene regulation network of flax through the genome-wide identification and characterization of microRNA and their potential targets [14,15]. However, with reference to transcription factors (TFs), the key players in gene regulation, the progress has been very limited in flax so far. This can be addressed by genome-wide identification of flax TFs, and investigation of their roles in gene regulatory networks will help to underpin the important biochemical, molecular and developmental programs of flax.

By application of embryo dissection methods [16,17,18,19], we systematically investigated flax-seed development and established an expressed sequence tag (EST)-based flax transcriptome database that identified 30,640 unigenes and gained new insights into gene activities associated with the developmental, physiological and biosynthetic processes involved in the synthesis of important seed constituents in flax [20]. Here, to further enrich and strengthen genomics resources in flax, we used high-throughput RNA-Seq to analyze the gene expression profiles across multiple tissues, including anther, root, ovary, mature seed and five stages of embryonic development in flax. Bioinformatics and statistical analyses of these transcriptome datasets identified gene co-expression modules and discovered diverse genes controlling biological processes, especially associated with essential genes for embryo development and accumulation of storage reserves in flax seeds. Taken together, the in-depth transcriptome datasets developed in this study will serve as an important genomics resource for the discovery and characterization of genes conferring useful traits in flax crops. 

## 2. Methods

### 2.1. Plant Growth Conditions and Tissue Collection

Breeder seed (F11) of *Linum usitatissimum* cv CDC Bethune, a medium-late-maturing oilseed flax developed by Crop Development Centre (CDC), University of Saskatchewan, was selfed for 7 generations (F18) as a single plant descendent in the Phytotron at the University of Saskatchewan. F19 seeds were germinated and grown in a growth chamber using a daily cycle consisting of 16 h of light (23 °C) and 8 h of dark (16 °C). Tissue collections were performed as previously described [16]. Each replicate of root, seed, anther and ovary samples was collected and combined from more than 10 individual plants, and 50–100 developing flax embryos were dissected at different development stages to isolate total RNA. 

### 2.2. RNA Extraction and High-Throughput Sequencing

Total RNA was extracted from each collected sample following the RNAqueous kit protocol (Ambion, Catalog# 1912) and RNAqueous-Micro kit protocol (Ambion, Catalog# 1931). The sample homogenization was performed using polypropylene pestles in 1.5-mL Eppendorf tubes after adding lysis buffer. The whole homogenization process was performed on ice. For RNA-seq profile analysis, Illumina mRNA-seq libraries were prepared using the TruSeq RNA kit (ver. 1, rev A) according to the manufacturer’s instructions. For conducting quality control (QC), an Agilent 2100 Bioanalyzer was used for quantification and quality determinations of sample libraries. For Illumina HiSeq 2000 sequencing (with an Illumina GA II instrument), four indexed libraries were pooled per sequencing lane, and paired-end sequencing was performed.

### 2.3. Mapping of RNA-seq Read to Reference Genome and Analysis of Expressed Genes

RNA-seq reads, obtained from 18 samples of different tissues and stages, were first preprocessed by trimming the adaptor sequences, filtering low-quality reads and eliminating short reads using Trimmomatic [21], with the argument ILLUMINACLIP:TruSeq3-SE:2:30:10 SLIDINGWINDOW:5:20 MINLEN:75. *Linum usitatissimum* genome released in 2012 and the corresponding annotation [2] from Phytozome v13 (https://phytozome.jgi.doe.gov/, accessed on 29 July 2022) were used as references for the analysis of clean reads alignment using STAR [22]. FeatureCounts [23] was used to generate gene-level counts with default parameters. The expression matrixes based on gene counts were transformed to normalized counts through DESeq2 [24], and variance stabilizing transformation (VST) was further performed. The expressed genes were determined based on the global expression of each gene across all stages/tissues in which it was expressed more than 10 normalized counts in at least one stage/tissue. 

### 2.4. Principal Component Analysis (PCA), Hierarchical Clustering, and Sample Distance 

Relative relatedness and reproducibility among biological replicates were examined by PCA using R FactoMineR package. Global comparisons of relative relatedness of samples from different developmental stages during embryogenesis and different tissues were performed using PCA based on the expressed genes defined above. Bi-plots of PC1 & PC2 were plotted using R ggplot2 package, and the explained variance of the principal component was calculated using the get_eigenvalue function in R FactoMineR package.

Hierarchical clustering was performed using the R ape package based on the expressed genes used in PCA. Pairwise sample distances between all samples were calculated based on Spearman’s correlation coefficients using the R dist function. Heatmaps revealing sample distance and hierarchical clustering were generated using the R pheatmap package.

### 2.5. Gene Co-Expression Analysis and WGCNA Network Construction

To identify genes that exhibit similar expression across different tissues/stages, VST values of expressed genes from all replicates were used to conduct weighted gene co-expression network analysis (WGCNA) [25]. Groups of closely connected genes with similar expression patterns were identified as gene modules. A soft thresholding power was determined by pickSoftThreshold function in the package and the “signed hybrid” network model was used. Hierarchical clustering was performed based on the topological overlap matrix and cutting the resulting dendrogram with the dynamicTreeCut program. Final network construction was built using blockwiseModules function with minModuleSize = 30, MaxModuleSize = 5000, mergeCutHeight = 0.2. Gene modules with stage-specific patterns of gene expression (specifically, patterns where gene expression was relatively high in one tissue/stage, and low in the others) were correlated with the specific expression patterns in different samples, based on significantly high Pearson correlation coefficient values (r > 0.8, *p*-value < 0.001). Based on the resulting adjacency matrix, we calculated the topological overlap, a concept defined for weighted networks, which is a robust and biologically meaningful measure of network interconnectedness. Line plots of gene expression patterns in each gene module were generated based on module eigengene (ME) values of all samples from different tissues/stages.

### 2.6. Identification of Tissue-Specific Expressed Genes and Functional Classification

Dominant gene expression in different tissues and embryo developmental stages was determined by both WGCNA modules with specific expression patterns and Tau index [26]; all replicates were considered. All specific genes were clustered together to plot the heatmap using the R pheatmap package, and Z-score transformation was applied prior to heatmap plotting.

Putative function, Gene Ontology (GO) and Kyoto Encyclopedia of Genes and Genomes (KEGG) pathway enrichments were predicted based on their corresponding Blast best-hit in *Arabidopsis* using the R topGO and KEGGprofile packages. The following arguments were applied in the Blast process, -evalue 1e-5 -best_hit_score_edge 0.05 -best_hit_overhang 0.25 -outfmt 7 -max_target_seqs 1 -max_hsps 5. Enrichment of GO and KEGG pathways was performed using the R clusterProfiler package. All expressed genes were used as background. REViGO analysis (http://revigo.irb.hr, accessed on 29 July 2022) was used to slim the enriched GO terms based on the “medium similarity” parameter. 

### 2.7. Gene Correlation and Transcription Factor Regulation Analysis in Different Gene Categories

The gene lists, including embryo-development-essential genes, storage proteins, carbohydrate- and lipid-related genes, fatty-acid synthesis and modification genes, and transcription factors (TFs), were identified in flax based on their homologs in *Arabidopsis* and the released *Arabidopsis* database (KEGG and Mapman) [27,28]. Expression patterns of members from these gene lists across tissues and developmental stages during embryogenesis were examined and clustered. A heatmap of different clusters was generated using R pheatmap package with log2 transformed or Z-score transformed normalized counts. Putative targets regulated by different TFs were predicted by GENIE3 [29] using TFs as query regulators, with the following arguments: tree.method = “RF”, ntrees = 1000, threshold = 0.005; obtained targets were recorded. 

## 3. Results

### 3.1. Transcriptome Profiling of Flax

Our previous work established an EST-based flax transcriptome covering seed and vegetative tissues [20]. To further advance detailed transcriptome profiles of important flax tissues with emphasis on the reproductive phases of embryogenesis and mature seeds, we performed high-throughput RNA-sequencing (RNA-seq) of embryos involved in key stages of embryogenesis (globular embryo, GE; heart embryo, HE; torpedo embryo, TE; cotyledon embryo, CE; mature embryo ME), mature seed, anther, ovary, and root, using the cultivar Bethune. To obtain a comprehensive global transcriptome landscape covering these representative developmental stages and tissues, we used the Illumina Hi-seq platform with libraries prepared from poly(A)+ transcripts. In total, 746.3 million paired-end reads were generated and aligned to the genome from Phytozome v13 as the reference sequence, of which 591.3 million reads were uniquely mapped. Via digital gene expression analysis, a total of 37,628 expressed genes were identified out of ~43,000 predicted protein coding genes in the flax genome (Supplemental Dataset S1).

### 3.2. Relationship of the Transcriptomes in Different Developmental Stages of Flax 

**Figure 1:**
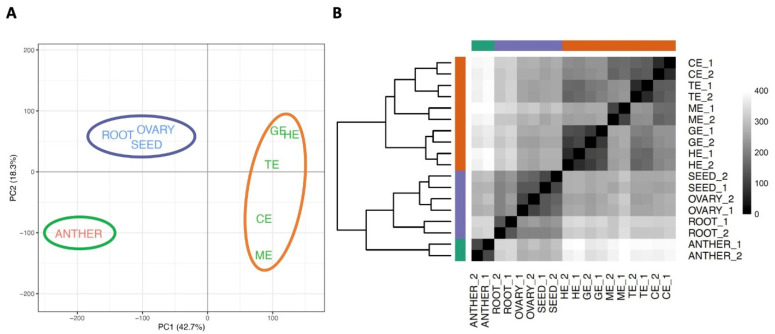
Relationship of the transcriptomes in different tissues and stages during embryonic development in flax. (**A**) Principal component analysis (PCA) using expressed genes in 9 tissues/stages in flax. *X*-axis, PC1; *Y*-axis, PC2. The proportion of variance for each principal component is indicated in brackets of axis titles. Three separated groups are labeled with different colors and in different ovals. GE, globular embryo; HE, heart embryo; TE, torpedo embryo; CE; cotyledon embryo; ME, mature embryo. (**B**) Heatmap of sample distance from different tissues/stages with two replicates (numbered 1 and 2). The grayscale spectrum represents sample distance calculated by R dist function ranging from 0 (black) to 400 (white), indicating high to low correlations, respectively. The hierarchical tree generated using expressed genes in each sample is shown on the left of the heatmap.

For a global view of gene expression between tissues of different flax developmental, we applied multiple clustering approaches, including principal-component analysis (PCA; Figure 1A, Supplemental Figure S1), hierarchical clustering, and pairwise sample distance (Figure 1B). These approaches were also used to measure the reproducibility among biological replicates. The findings from this analysis showed a clear clustering of replicate samples representing the same developmental-stage tissues. Then the relatedness among all samples was analyzed, identifying the three clearly separated groups, with the anther as the first group; root, mature seed, and ovary in the second group; and all embryonic development tissues in the third group (Figure 1A), indicating the spatial diversity in gene-expression programs in flax development. For embryo samples, the analysis showed a higher degree of correlation in neighboring progressive stages of embryo development (Figure 1B), and displayed a continuous transition of transcriptomes during embryonic development (from GE to HE to TE to CE to ME, Figure 1A), indicating the dynamic regulation of gene expression during embryogenesis. Moreover, the sample distance analysis among different stages of embryonic development divided all embryo samples into three subgroups, including middle stage (GE and HE), late stage (TE and CE), and mature stage (ME), and this assigned grouping is consistent with previous studies from other model plants and crop species [17,19,30]. The results from all three clustering analyses reveal close similarity amongst the transcriptome datasets and support substantial conservation of gene expression during embryogenesis in flax.

### 3.3. Gene Co-Expression Networks in Flax

**Figure 2:**
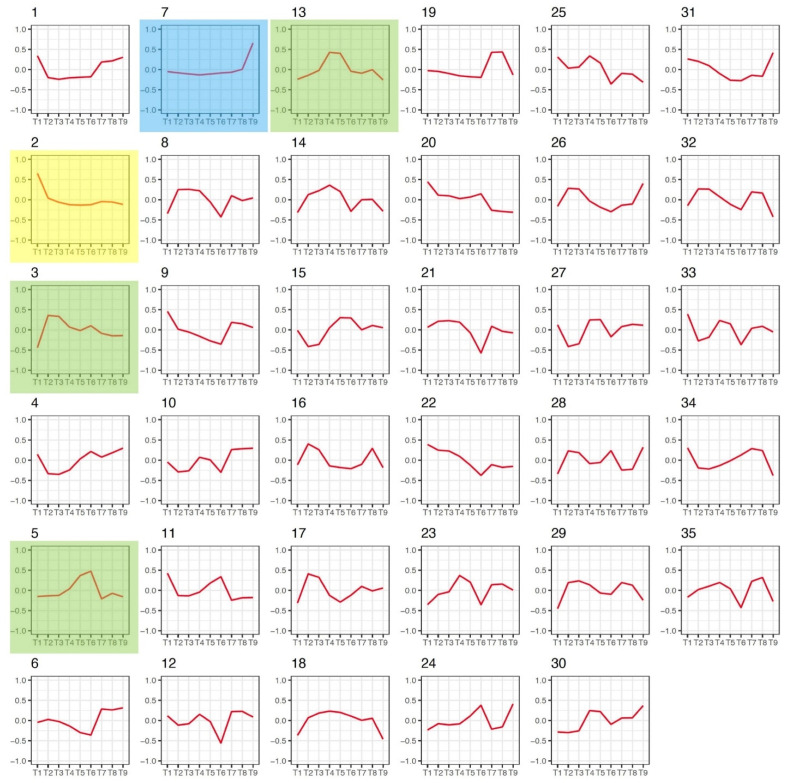
The 36 co-expressed gene modules identified through WGCNA. Module eigengene (ME) values were calculated for each tissue/stage. Line plots were generated based on ME values for all 36 modules. *X*-axis, nine tissues and stages. T1, anther; T2, GE; T3, HE; T4, TE; T5, CE; T6, ME; T7, ovary; T8, seed; T9, root. *Y*-axis, ME values. Five modules containing anther-specific (ME2), root-specific (ME7), and embryo-specific genes (ME3, 5, 13) were colored with yellow, blue, and green backgrounds, respectively.

Differential regulation of genes in corresponding tissues and dynamic expression patterns in distinct developmental stages are important indicators of corresponding biological pathways and processes. To determine the relative correlation of genes expressed in multiple tissues and different stages of embryogenesis, we totally identified 36 modules (clusters) based on 37,628 expressed genes from our RNA-seq datasets using weighted gene co-expression network analysis (WGCNA) [25] (Supplemental Figure S2). The dynamic hierarchical clustering approaches based on WGCNA define each gene cluster of co-expressed genes that follow specific patterns of expression. Then, for 36 modules, we obtained 36 diverse clusters of genes distinguished by the expression covering four different tissues and five embryonic developmental stages (Figure 2). Notably, two gene modules displayed anther-specific (module 2) and root-specific (module 7) profiles, providing dominantly expressed gene lists specifically for these two tissues (Supplemental Dataset S2). Three gene modules (ME 3, 5, and 13) showed embryo-specific patterns, in which members of Modules 3, 5, and 13 were dominantly expressed in the early, middle and late stages of embryogenesis, respectively (Figure 2 and Supplemental Dataset S2). 

For all modules, Pearson’s correlation coefficient values (r) between MEs and each tissue or stage were then used to determine the relationship between a module and a specific tissue or stage (Supplemental Figure S3). Consistently, Modules 2 and 7 (see arrows, Supplemental Figure S3) showed significant correlation with anther and root, respectively (r > 0.9 and *p*-value < 0.001), further confirming that the genes in these two modules are specific for these two tissues. By employing correlation analysis (Supplemental Figure S3), we further identified several embryo-dominant modules covering multiple stages defined by a dominant expression pattern in two or more consecutive stages of embryogenesis (r of each stage > 0.1, sum(r) > 0.9), including MEs 3, 5, 13, 14, 18, and 35. Besides MEs 3, 5, and 13, which displayed specific patterns in early, middle, and late stages of embryogenesis, as shown in Figure 2. MEs 14, 18 and 35 exhibited constitutive expression over all embryo developmental stages as well as in ovary and seed tissues. The gene members in these two modules represent housekeeping genes during the development and maturation of seeds. Further investigation of these genes may help to identify conserved regulatory networks of the seed development in flax. Moreover, there are no tissue-specific expression patterns identified for the remaining modules, indicating the likely complexity in the regulatory mechanism of these gene clusters. Taken together, our co-expression analysis discovered not only the specific genes for different tissues, but also identified gene clusters with similar expression patterns, thus providing a framework for exploring the underpinning key biological processes in flax.

### 3.4. Dominantly Expressed Genes and Biological Processes in Different Tissues of Flax

**Figure 3:**
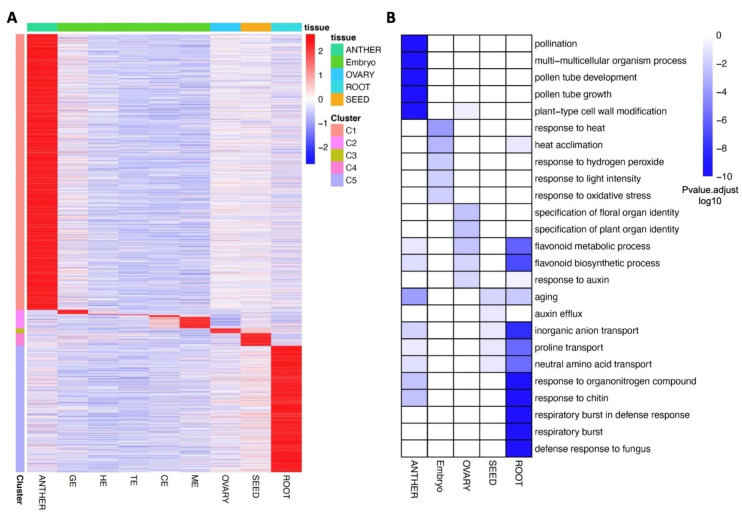
Dominant expressed genes in different tissues. (**A**) Heatmap of expression dynamics of gene members in five clusters (C1 to C5) representing tissue-specific expressed genes is shown. Genes in each cluster are associated with a color on the left of the heatmap. Different tissues and clusters are shown in different colors, as defined in the right inset. Z-score was applied for each row. For specificity, C1, anther; C2, embryo; C3, ovary; C4, seed; C5, root. (**B**) GO enrichment (biological process, BP) for each dominant gene cluster. A visual representation of GO enrichment for the genes in five tissue-specific clusters with enriched GO terms in the biological process category (on the *y*-axis) was plotted against each cluster (*x*-axis). The GO terms with a significant (*p* < 0.05) are shown and *p*-values are associated with colors shown in the right inset, from white (high) to blue (low).

The identification of specific and dominantly expressed genes in different tissues should provide a resource for understanding their respective divergence, crosstalk, and functions between these tissues. With WGCNA analysis, we further calculated the Tau Index, a widely used method for specific expression analysis [26], for each gene, to determine whether it was dominantly (Tau > 0.8 in any tissue) or broadly expressed. The genes identified by the Tau index were highly consistent with the tissue-specific genes in the modules identified through WGCNA. Only genes identified by both methods were defined as the dominantly expressed genes (Supplemental Dataset S3). However, the anther tissue contained the highest number of dominantly expressed genes, followed by root, embryo, mature seed, and ovary tissues (Figure 3A). 

The dominantly expressed genes in five different tissues formed five clusters, respectively. The gene ontology (GO) enrichment analysis and pathway analysis were performed for each cluster to investigate the putative biological processes associated with different flax tissues (Figure 3B, Supplemental Figure S4). For anther samples, significant GO terms involved in pollination, pollen tube development, and growth were enriched; and for ovary, significant GO terms involved in the specification of plant/floral organ identity were enriched, consistent with the representative biological processes for these two reproductive tissues. In embryo and root, defense-related GO terms were significantly enriched, where only abiotic stress response terms, including response to heat, hydrogen peroxide, and oxidative stress, were enriched in the embryo, but both abiotic and biotic stress response terms, including response to chitin, fungus, and respiratory burst, were enriched in root. Moreover, several transport-related terms, including proline transport, inorganic anion transport, and neutral amino acid transport, were enriched in anther, mature seed, and root tissue, indicating the potential tissue-specific long-distance transport in these tissues. Further analysis of these genes in different clusters through the Kyoto Encyclopedia of Genes and Genomes (KEGG) was performed to identify tissue-specific pathways. The significantly enriched pathway ontology (PO) terms are detailed in Supplemental Figure S4, where starch and sucrose metabolism, glucosinolate biosynthesis, flavonoid biosynthesis, cyanoamino acid metabolism, and phenylpropanoid biosynthesis are specific PO terms in the anther, embryo, ovary, mature seed, and root tissues, respectively. These GO and PO terms provide further support for the functional annotation of dominantly expressed gene clusters of key biological processes and metabolic pathways in different tissues of flax.

### 3.5. Expression Profiles of Transcription Factors and Embryo-Development-Essential Genes in Flax

The dynamic expression patterns of genes reflect their roles in different phases of development. Grouping co-expressed transcription factors (TFs) into clusters/modules with an enrichment of genes showing similar expression patterns will help towards the identification of uncharacterized genes or processes in flax. To identify such expression shifts, we performed TF co-expression analysis with their corresponding targets using a comprehensive method based on GENIE3 tools [29]. In total, 2678 TFs were identified, covering 56 types across all flax tissues. For each TF, 1 to 305 potential target genes were predicted, in which a C2H2 type TF (Lus10041633) possesses the most potential targets, and all E2F type TFs have the highest mean number of potential targets per TF (41.5) (Supplemental Dataset S4). 

To identify spatial gene expression trends regulated by TFs in flax tissues, we focused on the TFs identified in the five tissue-specific clusters from Figure 3A (C1 to C5). Flax anther and root tissues contain the most tissue-specific TFs and potential target genes (>90%, Supplemental Dataset S4), where Lus10041633 and another C2H2 type TF (Lus10018696) regulate the most potential target genes in the anther and root, respectively, emphasizing the core role of this type of TF. In contrast to anther and root tissues, MADS-Box- and bHLH-type TFs and their potential targets are significantly enriched in the embryo, consistent with the findings of these two TFs’ functions in the embryo as observed in other species [31]. Thus, we predicted that the cooperative interaction between MADS and bHLH might also play a key role in regulatory networks controlling embryonic development in flax. Interestingly, we found several genes essential for embryo development among the target genes of these two TFs, further supporting their cooperative functional importance in embryogenesis. 

**Figure 4:**
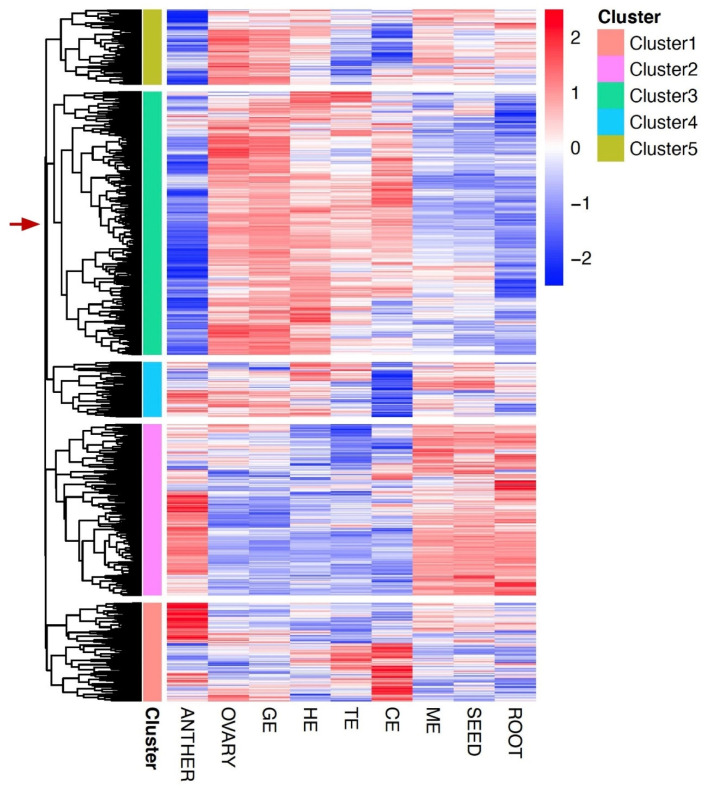
Cluster analysis of embryo-development-essential genes in flax. Heatmaps of clustered embryo-development-essential genes with similar expression patterns were generated. Five clusters were identified, shown with different colors as defined in the right inset. Z-score transformations of expression were performed for each gene across all tissues and stages of embryonic development. Expression levels were indicated by color scheme, from red (high) to blue (low) in corresponding tissues/stages, as defined in the inset. Genes in each cluster are associated with a color on the left of each heatmap3.6. Transcriptomic Regulation of Storage Reserve Genes in Flax.

In *Arabidopsis*, an updated dataset of 510 embryo-defective (EMB) genes, where loss-of-function mutations cause embryo defects, has been established recently to characterize and summarize the essential genes for embryo development [32]. Since we generated a dynamic transcriptome dataset for flax embryonic development over five stages in this study, it was possible to define the expressed essential genes for embryo development. Querying the *Arabidopsis* EMB mutant data sets through BLAST, an essential embryo developmental gene list covering 1072 expressed homologs in flax was developed (Supplemental Dataset S5). Based on the dynamic expression pattern across five stages of embryo development and other tissues, these essential genes for embryo development can be further grouped into five clusters through a hierarchical clustering approach (Figure 4). Notably, genes from Cluster 3 exhibit low expression levels in anther, root, and mature seed, but high expression levels during the embryonic developmental process and ovary. The expression pattern of Cluster 3 is consistent with the expected functions as the group of essential genes for embryo development, suggesting the important roles of its gene members during embryogenesis in flax. Particularly, two flax EMB genes (Lus10000126 and Lus10037831) were identified as the dominantly expressed genes at the mature embryo stage (Supplemental Dataset S3 and S5). This expression pattern is similar to their homologs in Arabidopsis (*ATG4* and *NOV*), suggesting the conserved functions of these two genes at the late stage of embryonic development [32].

**Figure 5:**
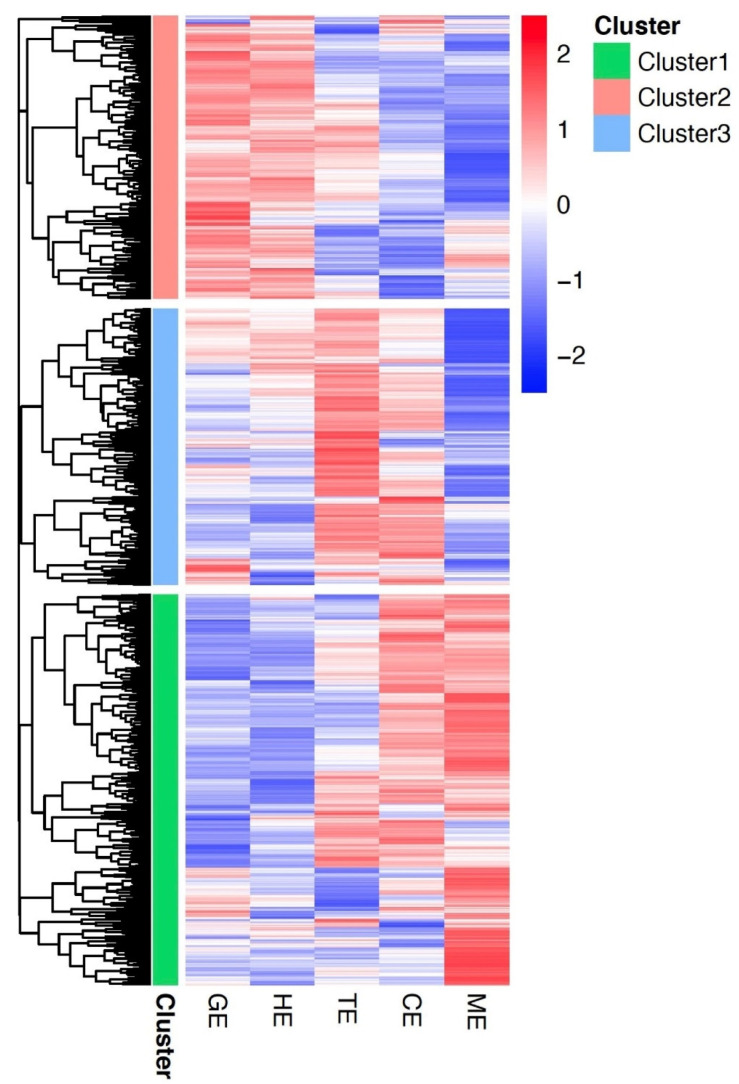
Storage-related lipid genes and carbohydrate gene-expression patterns during embryonic developmental process in flax. Heatmaps of clustered storage-related lipid and carbohydrate genes with similar expression patterns were generated during embryogenesis. Three clusters for each tissue were identified, shown with different colors as defined in the right inset. Z-score transformations of expression were performed for each gene across all developmental stages. Expression levels were indicated by color scheme, from red (high) to blue (low) in corresponding stages, as defined in the inset. Genes in each species or each cluster are associated with a color on the left of each heatmap. Three clusters represent three categories highly expressed in different adjacent stages.

To systematically explore the expression pattern divergence of storage-reserve-related genes in flax, we focused on the genes closely linked to the oil traits, and defined the expression characteristics of these genes during embryo development. To create a complete gene list containing all storage-reserve-related genes, the homologs in flax from three key categories, including storage protein-, carbohydrate-, and lipid-metabolism-related genes, were identified through querying the corresponding *Arabidopsis* database (Supplemental Dataset S6). Then the co-expression analysis using these storage reserve genes was performed, and three major clusters based on their expression patterns over five stages of embryo development were identified (Figure 5). These three clusters exhibited the dominantly expressed pattern at early (GE and HE), middle (TE and CE), and late (CE and ME) stages, respectively, indicating the functional differentiation of storage-reserve-related genes during the embryogenesis. Further investigation of individual genes in different clusters will recognize the biosynthesis and metabolism of diverse metabolites in flax. 

**Figure 6:**
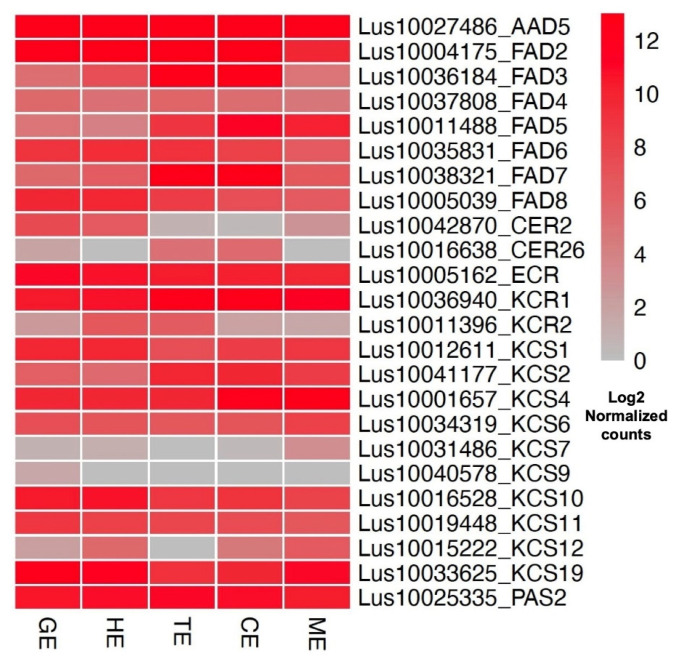
Expression of fatty-acid metabolic genes during embryonic developmental process in flax. Heatmap of the expression level of fatty-acid metabolic genes in five embryo developmental stages. Expression levels calculated using Log_2_ transformed normalized counts are indicated by color gradient, from red (high) to grey (low) in the five stages of embryogenesis.

As an important oil crop, the fatty-acid (FA) composition of storage reserves in flax is an important trait for targeted improvement. To gain insights into the expression patterns of FA-related genes during embryogenesis, we extracted all known genes involved in FA biosynthesis and modification from the flax genome by querying the corresponding *Arabidopsis* Mapman4 and KEGG database (Supplemental Dataset S6). After removing the significantly lower expressed genes, the putative FA-biosynthesis- and modification-related genes in flax were identified and their expression patterns were plotted in Figure 6. Most of them are highly expressed in embryo, consistent with their role in the seed oil biosynthetic process. The expression of several fatty-acid desaturase (*FAD*) genes showed an increase from GE to ME during embryogenesis (e.g., *FAD3*, *FAD5*, *FAD7*), suggesting coordinated accumulation of unsaturated linoleic acid and linolenic acid at the middle and late stages of embryo development in flax. 

## 4. Discussion

Uncovering gene expression patterns over dynamic seed developmental stages and diverse other key tissues will help to define the impact of regulatory networks associated with the agricultural traits in flax. Previous transcriptome studies in flax have used EST-based approaches [20], which are not as quantitative for clearly defining gene expression compared to more in-depth RNA-seq based studies. The datasets developed in this present study employed RNA-seq methods to overcome this limitation. Additionally, the tissue-specific expressed gene sets developed in this study contributed immensely to the investigation of tissue-specific functionality changes. Co-expression analysis grouped all expressed genes and TFs into different modules based on their expression patterns across different tissues and stages, providing a detailed list for further screening of co-expressed interactive genetic factors involved in important agricultural traits. Moreover, this analysis was applied in two important biological processes of flax development: essential development for embryo and storage-reserve accumulation, identifying the homologous genes of these processes in flax and revealing their expression behaviors in comparison to other plant species. Taken together, our study provides advanced insights into the regulatory programming of the transcriptome by revealing dynamic expression patterns and their associated functional transitions over different embryo developmental stages in flax.

Annotated genes associated with essential embryo development in flax were identified in the present study (Supplemental Dataset S5) through querying the *Arabidopsis* EMB mutant datasets [32]. These essential embryo-development genes showing similar expression patterns were assembled into five major clusters. The final essential embryo development gene list contains the genes from Cluster 3 (Figure 4), that display moderately dynamic pattern with high expression levels. This cluster contains 426 genes, and most of them show high gene expression at globular embryo stage. In *Arabidopsis*, most EMB mutants show lethality due to their essential functions at globular or heart stages [32]. Therefore, some of the EMB homologs in Cluster 3 with high expression at globular stage likely represent these essential and important functions. We found that most of these EMB genes encode proteins that function in protein synthesis or RNA binding and modification (Supplemental Dataset S5), as probably required in chloroplast protein translation at globular stage in flax, consistent with the finding in *Arabidopsis* [33]. By contrast, some EMB homologs in Cluster 4 showed higher expression in both anther and embryos. Functional analysis suggested that most of these EMB genes encode mitochondrial translation, transcriptional regulation, and signaling processes (Supplemental Dataset S5). Mutants defective in genes required for mitochondrial translation are often defective in gametophyte development [34]; thus, loss of function in these EMB genes might result in lethality to both gametophyte and embryo developmental programs in flax. Together, our dataset and analysis summarized the EMB-expressed genes in flax and grouped them into different categories based on their expression patterns across tissues. Understanding the relationship between protein function, genetic redundancy, and mutant phenotypes associated with the EMB genes in flax remains a challenge; however, our datasets could help to define the functions of EMB homologs in flax. 

The flax seed contains many nutritionally important products, such as proteins, fatty acids, lignans, flavonoids, and mucilage. In this study, we queried for genes involved in storage-reserve accumulation and fatty-acid biosynthesis and modification, and these analyses generated three gene clusters based on their expression patterns (Figure 5). Although most flax storage reserves are enriched in the middle and late embryo-developmental stages, around one-third of storage-reserve-related genes have their highest expression peaks at the early developmental stage (Figure 5 Cluster 2), indicating an early initiation of storage-reserve compound biosynthesis. In particular, GDSL esterases and lipases, an important subclass of lipolytic enzymes with multifunctional properties, were enriched in this cluster. The GDSL esterase/lipase gene family has been reported to be involved in multiple biological processes, such as response to nutrient deficiency, chemical and hormonal treatments, and biotic and abiotic stresses [35]. However, as knowledge of the GDSL esterase/lipase gene family in flax is limited, our datasets provide the dynamic expression pattern of this gene family and note down the key members involved in the early accumulation of storage reserves. Two homologs (Lus10014005 and Lus10015428) of an important TF, *Arabidopsis* B3 domain transcription factor LEC2, were also clustered in Cluster 2, with higher expression at early embryo developmental stages. LEC2 is a central regulator of embryogenesis in *Arabidopsis* that serves critical roles in the maintenance of embryo morphology, specification of cotyledon identity, suppression of premature germination, and fine-tuning of the lipid biosynthetic pathway in seeds [36,37]. In contrast to the expression pattern of LEC2 in *Arabidopsis* with high expressions in both early and late stages of embryonic development, two homologs of LEC2 in flax are highly expressed in the early stages of embryogenesis, indicating the different regulatory mechanisms of LEC2 in *Arabidopsis* and flax. This different expression pattern likely influences the morphology and composition of storage reverses between two species. Further investigation of flax LEC2 TF and its connection to the regulation of downstream storage reserve genes could aid targeted improvement of storage-reserve-related traits in flax. 

In flax, the FA profile in seed storage reserves is an important and desirable trait. To explore the expression patterns of FA-associated genes, in this study, we identified putative FA biosynthesis and modification-related genes in flax (Supplemental Dataset S6) and their dynamic expression pattern during embryogenesis. In *Arabidopsis*, fatty-acid desaturases (FADs) are fatty-acid modifying enzymes that introduce double bonds into fatty acyl chains. All important FAD homologs (*FAD2* to *FAD8*) in flax were highly expressed in the embryo samples (Figure 6). FAD3 of *Arabidopsis* encodes an endoplasmic reticulum localized enzyme responsible for the synthesis of 18:3 fatty acids from phospholipids; FAD5 encodes a chloroplastic enzyme responsible for the synthesis of 16:1 fatty acids from galactolipids and sulpholipids in *Arabidopsis*; FAD7 is a plastid-localized FAD involved in the synthesis of C16 and C18 polyunsaturated fatty acids in the prokaryotic glycerolipid pathway [38]. Expression of these three FADs shows an increase from GE to ME over the embryonic development in flax (Figure 6), in agreement with the expression patterns of their homologs in *Arabidopsis* and some crops [17,19,39] and the gradual accumulation of the ALA and LA in flax seed. Characterizing the molecular functions of each FAD gene family member in flax might provide additional insights to improve the important flax oilseed products ALA and LA. 

In some oil seed plants, C16 and C18 fatty acids can be modified through chain elongation to generate very long chain fatty acids (VLCFA), e.g., long-chain omega-3 eicosapentaenoic acid (EPA) and DHA docosahexaenoic acid (DHA). Genes encoding enzymes of the cytosolic fatty-acid elongase include β-ketoacyl-CoA synthase (KCS), β-ketoacyl-CoA reductase (KCR), β-hydroxyacyl-CoA dehydratase (HCD), and enoyl-CoA reductase (ECR). Interestingly, although VLCFAs are almost undetectable in flax seed, most of these genes’ homologs exist in the flax genome and are highly expressed during embryo development (Figure 6). The key fatty-acid elongation gene in *Arabidopsis* is *Fatty Acid Elongation 1* (*AtFAE1/KCS18*), a member of the KCS gene family that encodes the enzyme responsible for two condensation steps in the elongation from 18:1 to 20:1 and 22:1 in developing seeds [40]. Our analysis did not find homologs of *FAE1* in the flax genome, suggesting that the VLCFA biosynthesis pathway involving FAE1 function is likely replaced by other members of this gene family. This question is worth investigating and could be addressed by the integrative analysis based on the loss-of-function mutants of KCS gene family members in flax and the corresponding gene-expression profiles from our dataset.

Overall, we generated a comprehensive and in-depth transcriptome dataset covering multiple reproductive and vegetative flax tissues and different embryo developmental stages. Through detailed analyses of this dataset, we established the co-expression networks in flax based on their gene expression patterns, identified the associated TFs, and analyzed dominantly expressed genes in different stages and tissues of vegetative and reproductive development. Moreover, we identified storage-reserve-synthesis-related genes and essential genes in flax embryo development, and investigated their dynamic expression patterns. Together, our dataset and analysis provide a comprehensive valuable resource for studying seed development and seed-oil composition in flax.

## Data Availability

All RNA-seq raw data generated from this study have been deposited into NCBI BioProject under accession number PRJNA663265.

## Supplementary Materials

The following are available online at https://www.mdpi.com/article/10.3390/plants11152031/s1, Figure S1: Principal component analysis (PCA) of the transcriptomes for nine different tissues and stages in Flax. Figure S2: WGCNA modules (Clusters) dendrogram. Figure S3: Correlation relationship between modules and tissues. Figure S4: KEGG enrichment for each dominant gene cluster. Dataset S1: Log-transformed normalized counts from different tissues of flax. Dataset S2: Co-expressed modules identified from WGCNA. Dataset S3: Dominantly expressed genes in different stages and tissues in flax. Dataset S4: Identification of transcription factors (TFs) in flax. Dataset S5: Identification of embryo-defective (EMB) genes in flax based on Arabidopsis homologs. Dataset S6: Identification of storage-reserve-related genes in flax based on Arabidopsis database.

## References

[1] Oomah B.D. (2001). Flaxseed as a functional food source. J. Sci. Food Agric..

[2] Touré A., Xueming X. (2010). Flaxseed Lignans: Source, Biosynthesis, Metabolism, Antioxidant Activity, Bio-Active Components, and Health Benefits. Compr. Rev. Food Sci. Food Saf..

[3] Singh K.K., Mridula D., Rehal J., Barnwal P. (2011). Flaxseed: A potential source of food, feed and fiber. Crit. Rev. Food Sci. Nutr..

[4] Goyal A., Sharma V., Upadhyay N., Gill S., Sihag M. (2014). Flax and flaxseed oil: An ancient medicine & modern functional food. J. Food Sci. Technol..

[5] Ivanov S., Rashevskaya T., Makhonina M. (2011). Flaxseed additive application in dairy products production. Procedia Food Sci..

[6] Bastaki N.K., Cullis C.A. (2014). Floral-dip transformation of flax (Linum usitatissimum) to generate transgenic progenies with a high transformation rate. J. Vis. Exp. JoVE.

[7] Wang Z., Hobson N., Galindo L., Zhu S., Shi D., McDill J., Yang L., Hawkins S., Neutelings G., Datla R. (2012). The genome of flax (Linum usitatissimum) assembled de novo from short shotgun sequence reads. Plant J. Cell Mol. Biol..

[8] Dmitriev A.A., Kudryavtseva A.V., Krasnov G.S., Koroban N.V., Speranskaya A.S., Krinitsina A.A., Belenikin M.S., Snezhkina A.V., Sadritdinova A.F., Kishlyan N.V. (2016). Gene expression profiling of flax (Linum usitatissimum L.) under edaphic stress. BMC Plant Biol..

[9] Dash P.K., Rai R., Mahato A.K., Gaikwad K., Singh N.K. (2017). Transcriptome Landscape at Different Developmental Stages of a Drought Tolerant Cultivar of Flax (Linum usitatissimum). Front. Chem..

[10] Dash P.K., Cao Y., Jailani A.K., Gupta P., Venglat P., Xiang D., Rai R., Sharma R., Thirunavukkarasu N., Abdin M.Z. (2014). Genome-wide analysis of drought induced gene expression changes in flax (Linum usitatissimum). GM Crops Food.

[11] Zhang N., Deyholos M.K. (2016). RNASeq Analysis of the Shoot Apex of Flax (Linum usitatissimum) to Identify Phloem Fiber Specification Genes. Front. Plant Sci..

[12] Gui B., Shim Y.Y., Datla R.S.S., Covello P.S., Stone S.L., Reaney M.J.T. (2012). Identification and Quantification of Cyclolinopeptides in Five Flaxseed Cultivars. J. Agric. Food Chem..

[13] Xie D., Dai Z., Yang Z., Tang Q., Deng C., Xu Y., Wang J., Chen J., Zhao D., Zhang S. (2019). Combined genome-wide association analysis and transcriptome sequencing to identify candidate genes for flax seed fatty acid metabolism. Plant Sci. Int. J. Exp. Plant Biol..

[14] Zhang T., Li Z., Song X., Han L., Wang L., Zhang J., Long Y., Pei X. (2020). Identification and Characterization of microRNAs in the Developing Seed of Linseed Flax (Linum usitatissimum L.). Int. J. Mol. Sci..

[15] Barvkar V.T., Pardeshi V.C., Kale S.M., Qiu S., Rollins M., Datla R., Gupta V.S., Kadoo N.Y. (2013). Genome-wide identification and characterization of microRNA genes and their targets in flax (Linum usitatissimum): Characterization of flax miRNA genes. Planta.

[16] Xiang D., Venglat P., Tibiche C., Yang H., Risseeuw E., Cao Y., Babic V., Cloutier M., Keller W., Wang E. (2011). Genome-Wide Analysis Reveals Gene Expression and Metabolic Network Dynamics during Embryo Development in Arabidopsis. Plant Physiol..

[17] Gao P., Xiang D., Quilichini T.D., Venglat P., Pandey P.K., Wang E., Gillmor C.S., Datla R. (2019). Gene expression atlas of embryo development in Arabidopsis. Plant Reprod..

[18] Xiang D., Quilichini T.D., Liu Z., Gao P., Pan Y., Li Q., Nilsen K.T., Venglat P., Esteban E., Pasha A. (2019). The Transcriptional Landscape of Polyploid Wheats and Their Diploid Ancestors during Embryogenesis and Grain Development. Plant Cell.

[19] Gao P., Quilichini T.D., Yang H., Li Q., Nilsen K.T., Qin L., Babic V., Liu L., Cram D., Pasha A. (2022). Evolutionary divergence in embryo and seed coat development of U’s Triangle Brassica species illustrated by a spatiotemporal transcriptome atlas. New Phytol..

[20] Venglat P., Xiang D., Qiu S., Stone S.L., Tibiche C., Cram D., Alting-Mees M., Nowak J., Cloutier S., Deyholos M. (2011). Gene expression analysis of flax seed development. BMC Plant Biol..

[21] Bolger A.M., Lohse M., Usadel B. (2014). Trimmomatic: A flexible trimmer for Illumina sequence data. Bioinformatics.

[22] Dobin A., Davis C.A., Schlesinger F., Drenkow J., Zaleski C., Jha S., Batut P., Chaisson M., Gingeras T.R. (2013). STAR: Ultrafast universal RNA-seq aligner. Bioinformatics.

[23] Liao Y., Smyth G.K., Shi W. (2014). featureCounts: An efficient general purpose program for assigning sequence reads to genomic features. Bioinformatics.

[24] Love M.I., Huber W., Anders S. (2014). Moderated estimation of fold change and dispersion for RNA-seq data with DESeq2. Genome Biol..

[25] Langfelder P., Horvath S. (2008). WGCNA: An R package for weighted correlation network analysis. BMC Bioinform..

[26] Kryuchkova-Mostacci N., Robinson-Rechavi M. (2017). A benchmark of gene expression tissue-specificity metrics. Brief. Bioinform..

[27] Kanehisa M., Goto S. (2000). KEGG: Kyoto encyclopedia of genes and genomes. Nucleic Acids Res..

[28] Schwacke R., Ponce-Soto G.Y., Krause K., Bolger A.M., Arsova B., Hallab A., Gruden K., Stitt M., Bolger M.E., Usadel B. (2019). MapMan4: A Refined Protein Classification and Annotation Framework Applicable to Multi-Omics Data Analysis. Mol. Plant.

[29] Huynh-Thu V.A., Irrthum A., Wehenkel L., Geurts P. (2010). Inferring Regulatory Networks from Expression Data Using Tree-Based Methods. PLoS ONE.

[30] Hao Z., Zhang Z., Xiang D., Venglat P., Chen J., Gao P., Datla R., Weijers D. (2021). Conserved, divergent and heterochronic gene expression during Brachypodium and Arabidopsis embryo development. Plant Reprod..

[31] Di Marzo M., Roig-Villanova I., Zanchetti E., Caselli F., Gregis V., Bardetti P., Chiara M., Guazzotti A., Caporali E., Mendes M.A. (2020). MADS-Box and bHLH Transcription Factors Coordinate Transmitting Tract Development in Arabidopsis thaliana. Front. Plant Sci..

[32] Meinke D.W. (2020). Genome-wide identification of EMBRYO-DEFECTIVE (EMB) genes required for growth and development in Arabidopsis. New Phytol..

[33] Parker N., Wang Y., Meinke D. (2014). Natural Variation in Sensitivity to a Loss of Chloroplast Translation in Arabidopsis. Plant Physiol..

[34] Berg M., Rogers R., Muralla R., Meinke D. (2005). Requirement of aminoacyl-tRNA synthetases for gametogenesis and embryo development in Arabidopsis. Plant J. Cell Mol. Biol..

[35] Chepyshko H., Lai C.-P., Huang L.-M., Liu J.-H., Shaw J.-F. (2012). Multifunctionality and diversity of GDSL esterase/lipase gene family in rice (Oryza sativa L. japonica) genome: New insights from bioinformatics analysis. BMC Genom..

[36] Stone S.L., Kwong L.W., Yee K.M., Pelletier J., Lepiniec L., Fischer R.L., Goldberg R.B., Harada J.J. (2001). LEAFY COTYLEDON2 encodes a B3 domain transcription factor that induces embryo development. Proc. Natl. Acad. Sci. USA.

[37] Singer S.D., Jayawardhane K.N., Jiao C., Weselake R.J., Chen G. (2021). The effect of AINTEGUMENTA-LIKE 7 over-expression on seed fatty acid biosynthesis, storage oil accumulation and the transcriptome in Arabidopsis thaliana. Plant Cell Rep..

[38] Ohlrogge J., Browse J. (1995). Lipid biosynthesis. Plant Cell.

[39] Gao P., Quilichini T.D., Zhai C., Qin L., Nilsen K.T., Li Q., Sharpe A.G., Kochian L.V., Zou J., Reddy A.S.N. (2021). Alternative splicing dynamics and evolutionary divergence during embryogenesis in wheat species. Plant Biotechnol. J..

[40] James D.W., Lim E., Keller J., Plooy I., Ralston E., Dooner H.K. (1995). Directed tagging of the Arabidopsis FATTY ACID ELONGATION1 (FAE1) gene with the maize transposon activator. Plant Cell.

