# Supplementary material for: Spatiotemporal Transcriptomic Atlas of Developing Embryos and Vegetative Tissues in Flax
Source: Plants (Basel). 2022 Aug 4;11(15):2031. doi: 10.3390/plants11152031 (PMC9370790; doi:10.3390/plants11152031)
Supplement: Supplementary file 1 [file plants-11-02031-s001.zip › Supplemental Figures.pdf]

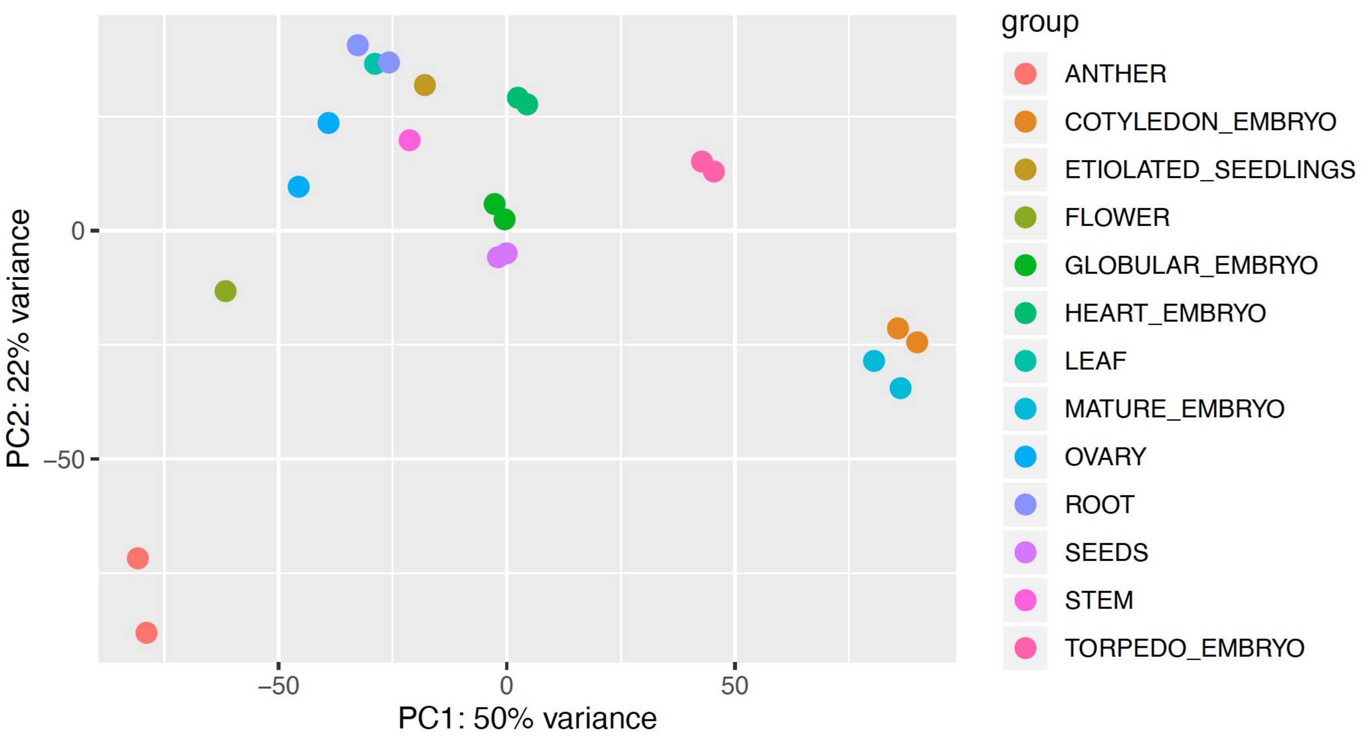

Supplemental Figure S1. Principal component analysis (PCA) of the transcriptomes for nine different tissues and stages in Flax. Biological replicates from the same tissue/stage are represented by the same color as defined in the right inset.

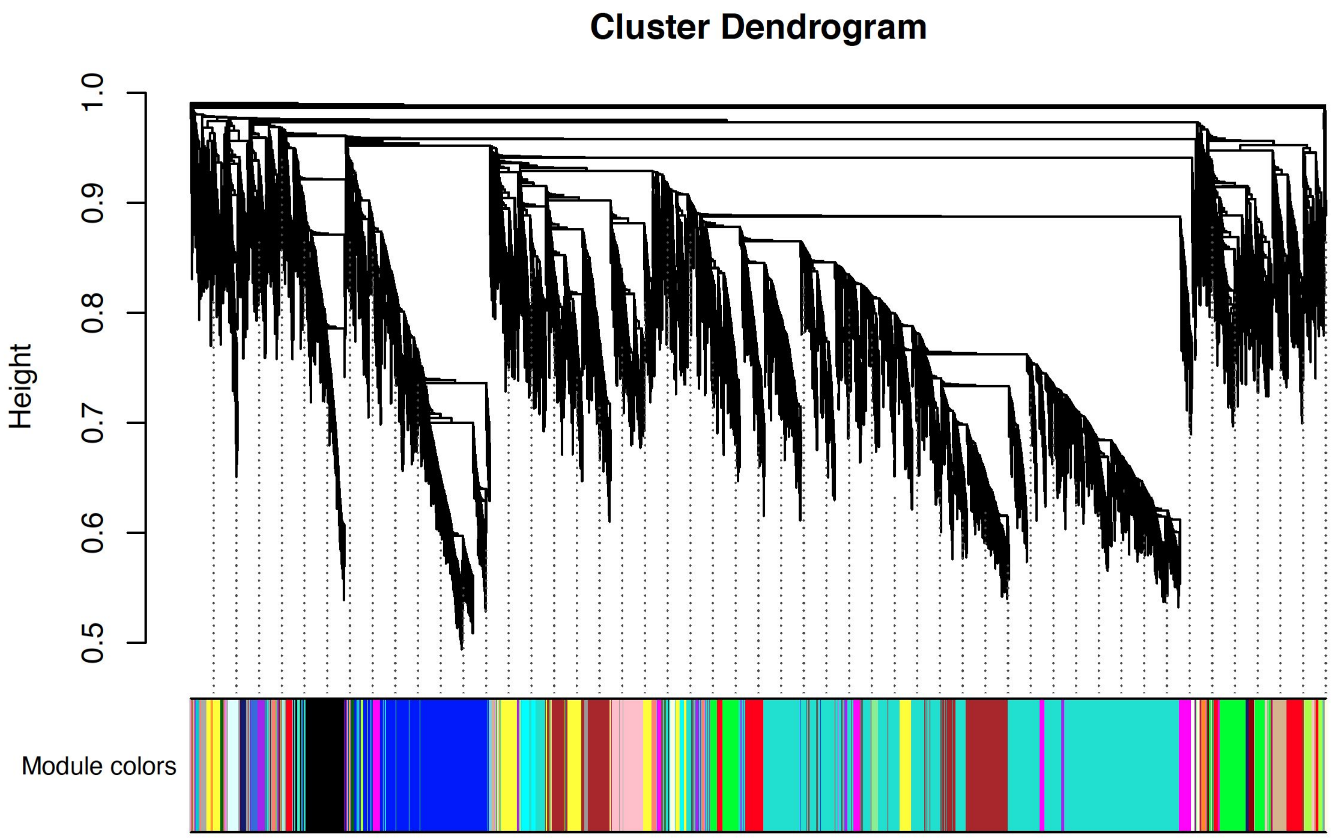

Supplemental Figure S2. WGCNA modules (Clusters) dendrogram. Hierarchical cluster tree showing modules of co-expressed genes, was identified with a total of 36 co-expressed gene modules identified. The height (y-axis) indicates level of correlation. Colors represent the 36 different modules, with gray indicating genes that could not be assigned to any module.

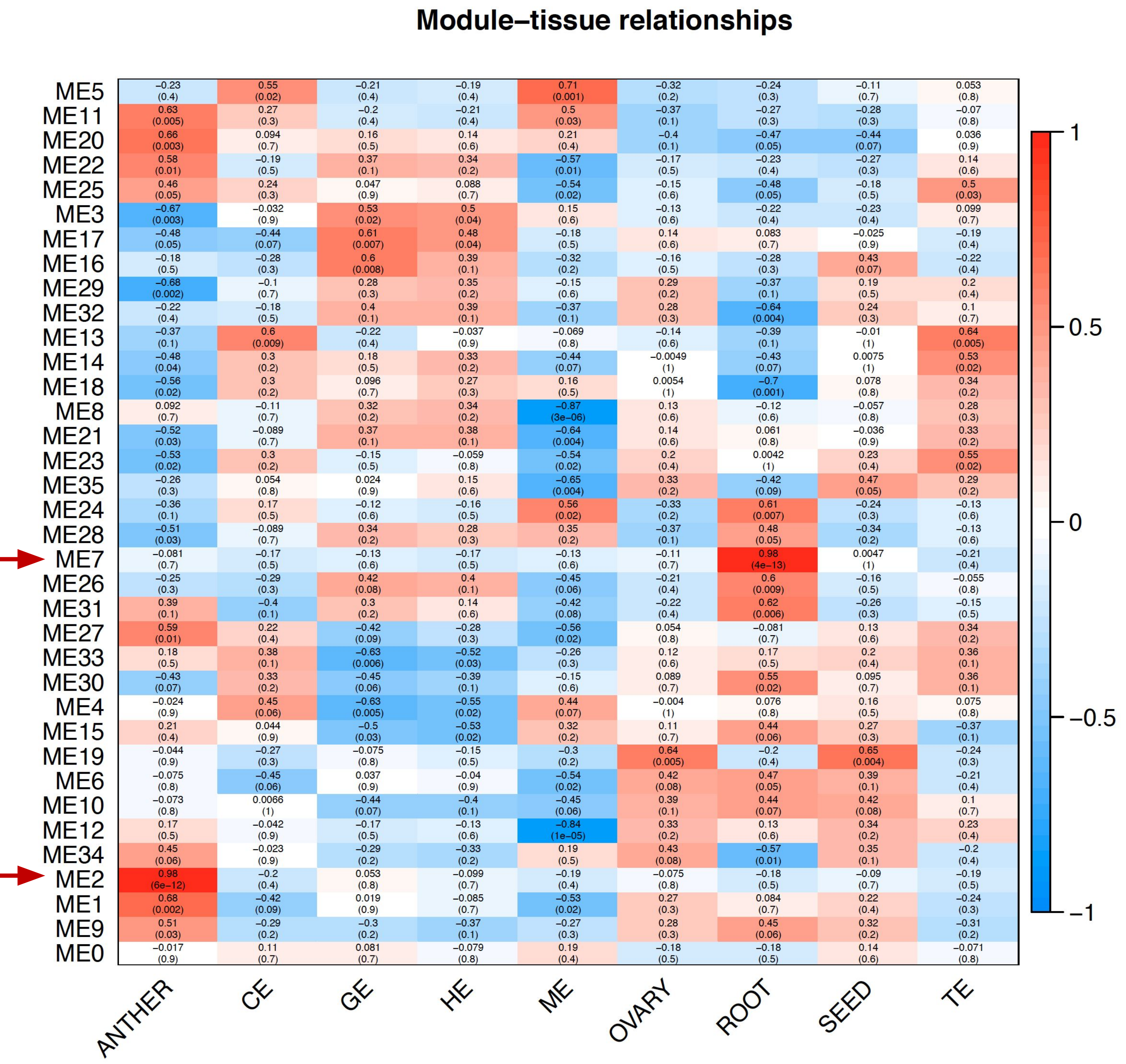

Supplemental Figure S3. Correlation relationship between modules and tissues. Heatmap of correlations between co-expressed gene modules and nine tissues and stages of embryo development, with numerical Pearson's correlation coefficient (top) and corresponding p-values (bottom). The color scheme, from red through white to blue, indicates the level of correlation, from high to low. Anther-specific (ME2) and root-specific (ME7) modules are identified by $r > 0.8$, $p < 0.001$ with red arrows.

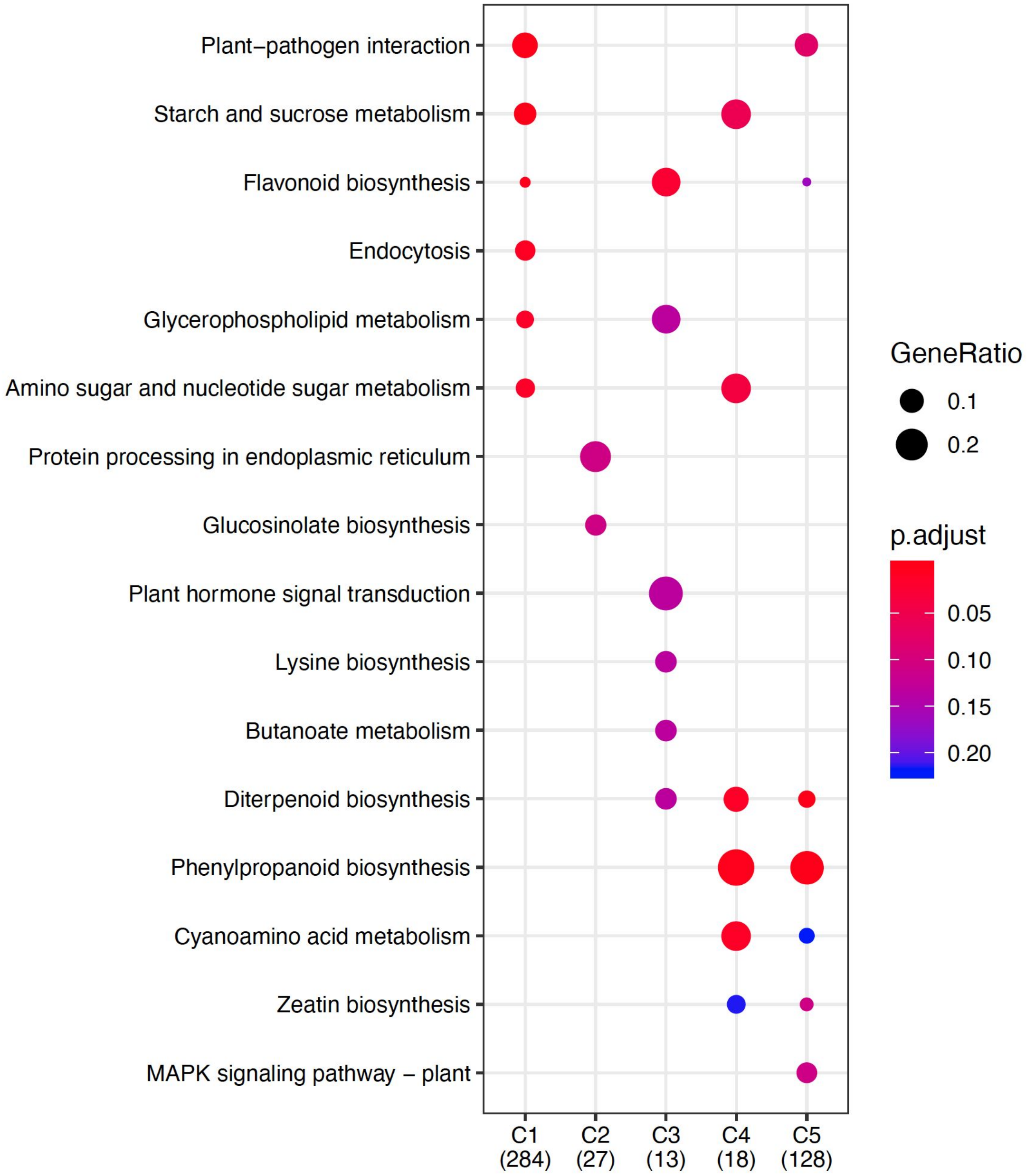

Supplemental Figure S4. KEGG enrichment for each dominant genes cluster. A visual representation of KEGG enrichment for the genes in five tissue-specific clusters was plotted against each cluster (x-axis). The KEGG terms with a significant p (< 0.20) are shown and p-values are associated with dot colors as defined in the right inset. Dot size represents related gene ratio in total genes of each cluster.
